# Palladium and Organocatalysis: An Excellent Recipe for Asymmetric Synthesis

**DOI:** 10.3390/molecules180910108

**Published:** 2013-08-22

**Authors:** M. Ángeles Fernández-Ibañez, Beatriz Maciá, Diego A. Alonso, Isidro M. Pastor

**Affiliations:** 1 Departamento de Química Orgánica, Facultad de Ciencias, Universidad Autónoma de Madrid, Cantoblanco, Madrid 28049, Spain; E-Mail: tati.fernandez@uam.es; 2 Division of Chemistry and Environmental Science, Faculty of Science and Engineering, Manchester Metropolitan University, John Dalton Extension, Oxford Road, Manchester M1 5GD, UK; E-Mail: b.macia-ruiz@mmu.ac.uk; 3 Departamento de Química Orgánica, Facultad de Ciencias and Instituto de Síntesis Orgánica (ISO), Universidad de Alicante, Apdo. 99, Alicante 03080, Spain

**Keywords:** palladium, organocatalysis, asymmetric synthesis, dual activation, tandem reaction, allylation, fluorination, decarboxylative protonation

## Abstract

The dual activation of simple substrates by the combination of organocatalysis and palladium catalysis has been successfully applied in a variety of different asymmetric transformations. Thus, the asymmetric α-allylation of carbonyl compounds, α-fluorination of acyl derivatives, decarboxylative protonation of β-dicarbonyl compounds, cyclization reactions of alkynyl carbonyl compounds and β-functionalization of aldehydes have been efficiently achieved employing this double-catalytic methodology.

## 1. Introduction

Organocatalysis has represented, in this new century, a breakthrough in the field of asymmetric organic synthesis [1], becoming an attractive complementary alternative for the well established transition-metal catalysis [2,3]. It was just a matter of time before both strategies were successfully combined for the improvement of catalytic processes [4,5,6]. The combination of organocatalysis and transition-metal catalysis allows not only the development of novel transformations, but also the enhancement of the stereochemistry, efficiency and/or scope of well known reactions [7]. One of the main challenges to overcome when merging these two systems, is tuning their compatibility to avoid negative interferences between them. Amongst transition metal catalysis, palladium-catalyzed reactions have gained a predominant place in the arsenal of synthetic chemists [2,8,9,10,11,12] and their combination with organocatalysts has resulted into very efficient cooperative dual systems. This review compiles the contributions of this cooperative dual catalytic system on asymmetric transformations.

## 2. Asymmetric α-Allylic Alkylation

**Figure 1:**
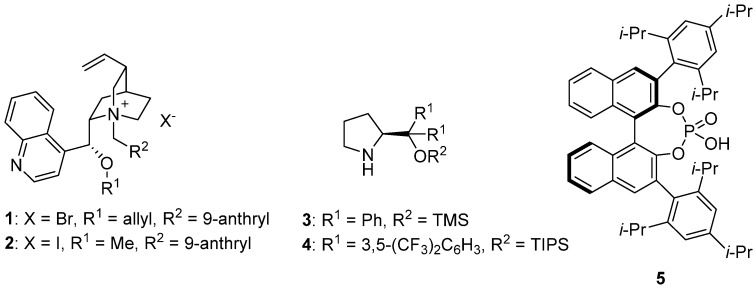
Chiral organocatalysts for α-allylation reactions.

The α-alkylation to carbonyl moieties is a classical reaction for the formation of carbon-carbon bonds in organic synthesis [13]. In particular, the asymmetric α-allylic alkylation stands out as a versatile methodology [14] where palladium complexes are of paramount importance, due to the easy generation of π-allyl intermediates as electrophiles [15,16]. Recently, α-allylation of carbonyl compounds has been achieved by merging the formation of π-allyl-palladium and the organocatalytic generation of nucleophilic species. In 2001, the asymmetric allylation of *tert*-butyl *N*-(diphenylmethylene)glycinate by the combination of the palladium complex [Pd(allyl)Cl]_2_, in the presence of PPh_3_ as ligand and salt **1** as chiral phase transfer catalyst (PTC) (Figure 1) was achieved, with a maximum ee of 61% [17]. The authors describe that the use of chiral bidentate ligands, such as BINAP, does not improve the outcome of the reaction, whilst the use of molecular sieves is crucial to obtain high enantioselectivities.

**Scheme 1:**
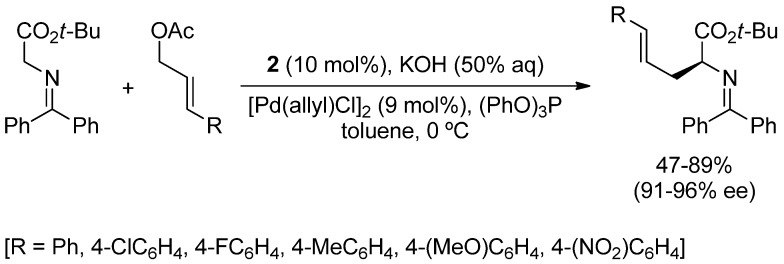
Allylic alkylation catalyzed by **2** and [Pd(C_3_H_5_)Cl]_2_/P(OPh)_3_.

Soon after, Takemoto and co-workers reported the allylation of glycinate derivatives with allylic acetates under similar reaction conditions: cinchonidinium salt **2** (10 mol%, Figure 1), [Pd(C_3_H_5_)Cl]_2_ (9 mol%), and P(OPh)_3_ (20 mol%) as ligand (Scheme 1) [18,19]. In this case, the use of a phosphite ligand is essential to achieve good enantioselectivities, which fall in the range 91%–96% ee. Indeed, the authors claim that more σ-donating ligands, such as phosphines, form a more reactive allyl-palladium complex which favors the reaction with the enolate in the absence of the chiral ion pair, and yields the corresponding product with poor enantioselectivity.

**Scheme 2:**
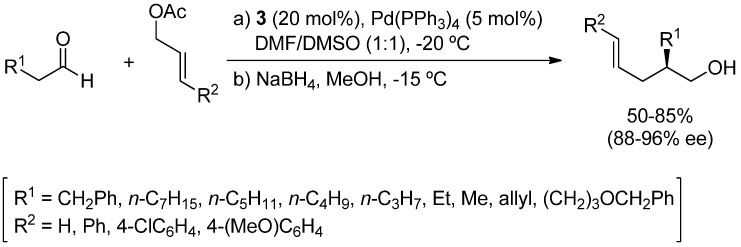
Allylic alkylation of aldehydes catalyzed by **3** and [Pd(PPh_3_)_4_].

The α-allylation of aldehydes by combination of an amine organocatalyst and a palladium complex, which generate a nucleophilic enamine and an electrophilic π-allyl palladium intermediate, respectively, has been described by the group of Córdova [20,21]. The co-catalyzed asymmetric allylic alkylations are carried out with the chiral pyrrolidine **3** (Figure 1) and [Pd(PPh_3_)_4_]. Thus, the corresponding alcohols, after in situ reduction of the allylated aldehydes, are obtained in high yields (50%–85%) and enantioselectivities ranging from 88% to 96% (Scheme 2). Interestingly, the sequence α-allylation of aldehydes and reductive amination allows the preparation of chiral amines in 41%–66% yield [21].

**Scheme 3:**
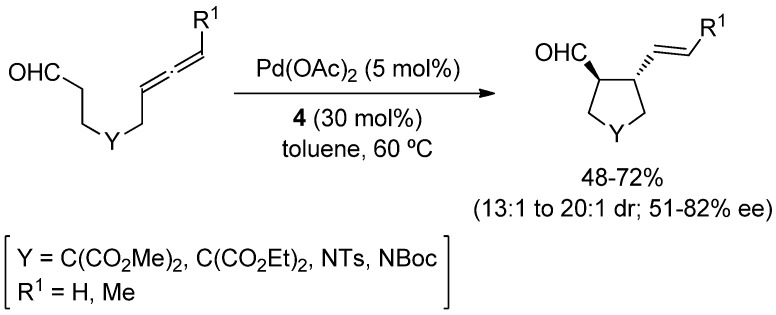
Amine/palladium co-catalyzed asymmetric cyclization.

More recently, the intramolecular version of this transformation has been reported with the formation of 5 and 6-membered rings starting from the corresponding 6- or 7-alkenal derivatives [22,23]; albeit an excess of an achiral secondary amine has to be employed [24] in combination with a chiral palladium complex, in order to obtain certain enantioselectivity. Contrary to this, the intramolecular cyclization of analogous allenic aldehydes, *i.e*., octa-6,7-dienal derivatives, was achieved using catalytic amounts of the chiral secondary amine **4** and Pd(OAc)_2_, without any phosphine ligand. This methodology provides the corresponding cyclopentanecarbaldehydes in good *trans*-diastereoselection (up to 20:1) and enantioselectivities up to 82% ee [25] (Scheme 3).

**Scheme 4:**
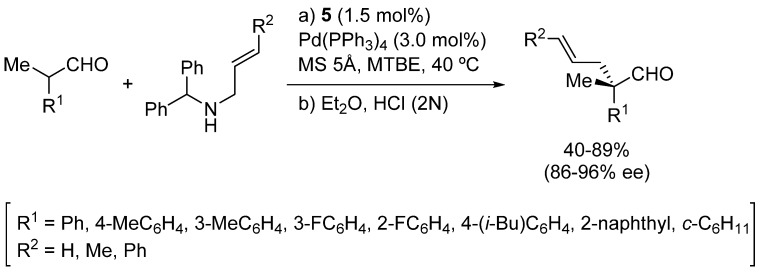
Enantioselective Pd/chiral phosphoric acid catalyzed α-allylation of aldehydes.

The group of List has reported the combination of a Pd(0) catalyst with the chiral phosphoric acid **5** as an effective formula for the enantioselective α-allylation of branched aldehydes with allylic amines [26] or allylic alcohols [27]. Thus, different aldehydes were reacted with *N*-benzhydrylallylamines in the presence of phosphoric acid **5** (1.5 mol%), Pd(PPh_3_)_4_ (3 mol%) and molecular sieves in *tert*-butyl methyl ether, to form the corresponding allylated aldehydes with α-quaternary stereogenic centers (Scheme 4) [26].

**Scheme 5:**
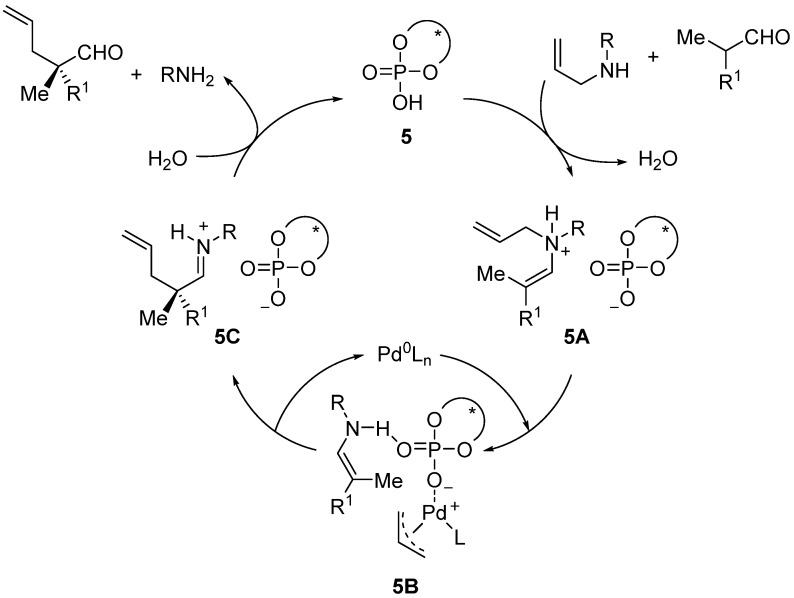
Proposed mechanism for the α-allylation of aldehydes in the presence of a chiral phosphoric acid and Pd(0) complex.

The authors suggest that the condensation of the aldehyde and the secondary amine in the presence of **5** produces the enamonium salt **5A**, which affords the intermediate **5B** in the presence of a Pd(0) complex (Scheme 5).

Finally, **5B** leads to the allylated iminium salt **5C** *via* nucleophilic attack of the enamine onto the π-allyl-palladium complex (Scheme 5). Regarding the use of allylic alcohols as allylating agents under the same reaction conditions, the results are not as satisfactory, and the expected products are obtained with lower enantiomeric excess (*c.a.* 10% ee), probably due to the formation of both *E*- and *Z*-enol isomers [27]. This problem can be overcome by using a secondary amine, such as benzhydryl amine (40 mol%), to form the corresponding enamine *in situ*. In this way, the α-allylation of aldehydes with allylic alcohols reaches similar levels of enantioselectivities as for the allylic amines [27].

## 3. Asymmetric α-Fluorination

**Figure 2:**
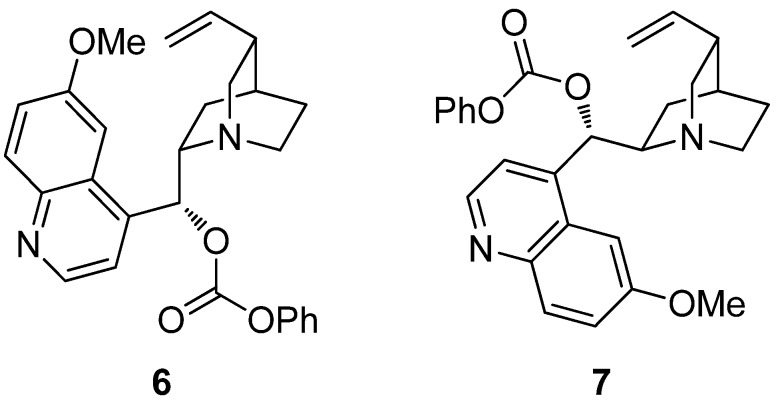
Benzoylquinine (BQ) (**6**) and benzoylquinidine (BQd) (**7**).

**Scheme 6:**
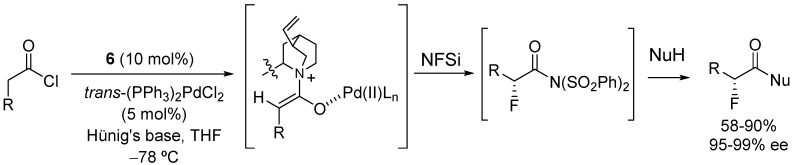
Catalytic enantioselective α-fluorination of acyl chlorides.

The catalytic asymmetric α-fluorination of acid chlorides has been shown by Lectka and co-workers to be a powerful method to synthesize a wide range of α-fluorocarboxylic acid derivatives in good yields and excellent enantioselectivities through electrophilic fluorination of a ketene enolate intermediate [28,29,30]. Lectka’s bifunctional catalytic system is based on the combination of the chiral nucleophiles benzoylquinine (**6**) or benzoylquinidine (**7**) and a transition metal Lewis acid cocatalyst, such as (PPh_3_)_2_PdCl_2_, in the presence of Hünig’s base. Under these conditions, the chiral ketene enolate intermediate reacts with *N*-fluorodibenzenesulfonamide (NFSi) to provide the corresponding α-fluoro bis(sulfonimide) intermediate, which can be quenched with a variety of nucleophiles to furnish α-fluorocarboxylic acids, amides, esters and even peptides (Scheme 6) [28]. The methodology is particularly attractive since both enantiomers of the product are available in similar selectivity by the selection of either benzoylquinine (**6**) or its pseudoenantiomer benzoylquinidine (**7**) (Figure 2).

**Figure 3:**
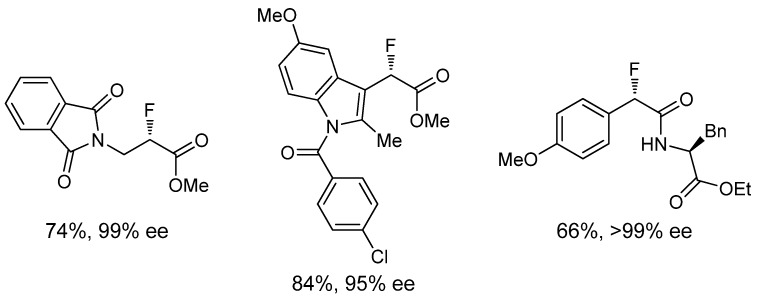
Representative examples of the asymmetric α-fluorination through a dual activation mechanism.

**Scheme 7:**
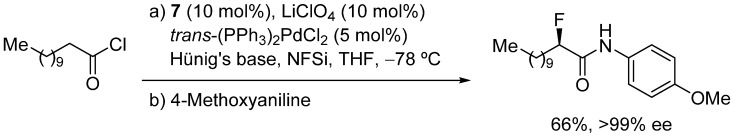
Catalytic enantioselective α-fluorination of an aliphatic acyl chloride.

**Figure 4:**
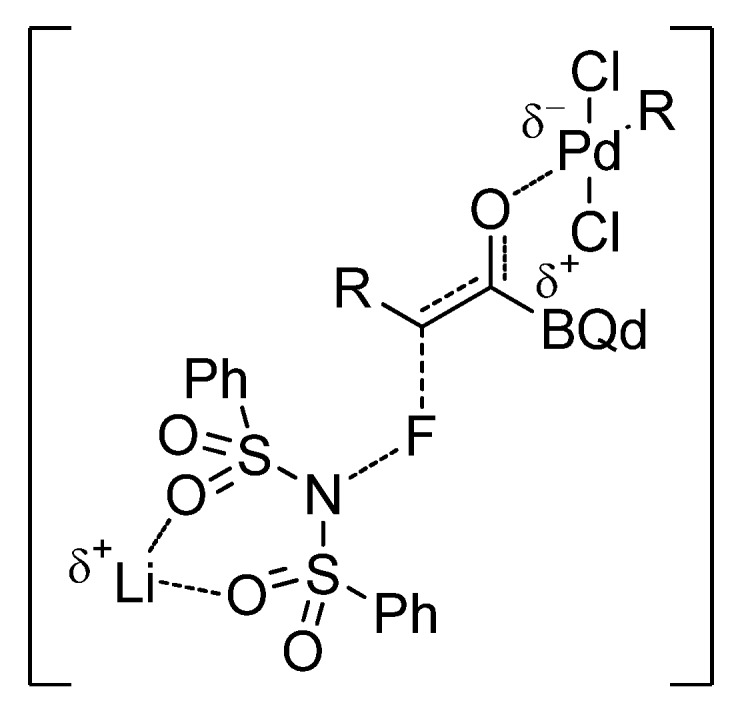
Proposed activation mode of the trifunctional catalytic system.

In addition, this dual activation strategy proved to be very efficient, in terms of yield and enantioselectivity, for 2-substituted aromatic and heteroaromatic acid chlorides with a variety of nucleophiles (Figure 3). However, the asymmetric α-fluorination of aliphatic acyl chlorides needs of a third catalyst, the alkali metal Lewis acid LiClO_4_ (10 mol%), to work cooperatively with the chiral nucleophile and the Pd catalyst and afford optically pure products in good yields (for a selected example, Scheme 7) [30]. Mechanistic studies have shown that the lithium salt activates the fluorinated agent; this input being necessary in the case of the less reactive aliphatic acyl chlorides (Figure 4).

**Figure 5:**
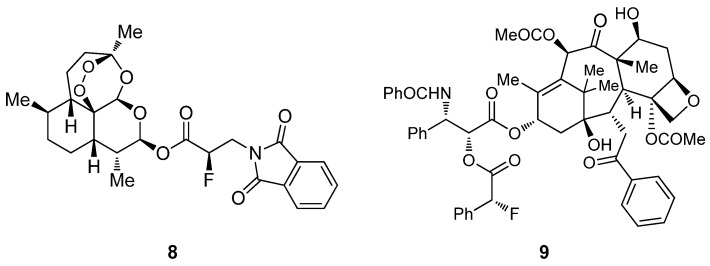
Fluorinated artemisinin derivative **8** and fluorinated taxol derivative **9**.

The α-fluorination of acid chlorides developed by Lectka *et al*. has been successfully employed for the site-specific functionalization of natural products and biologically active molecules with excellent diastereoselectivity [28,29]. For instance, quenching the fluorination reaction of 3-phthalimidopropionyl chloride with the lactol of the anti-malarian agent artemisinin, affords compound **8** in 75% yield and 81% ee (Figure 5) [29]. Also, the chemotherapeutic drug taxol, which has three distinct and potentially nucleophilic hydroxyl groups, reacted selectively by the secondary alcohol with the fluorinated intermediate of *p*-methoxyphenylacetyl chloride, affording compound **9** in 43% yield and 99% de (Figure 5) [29]. Using this strategy, Leckta has reported the synthesis of biologically relevant α-fluorinated carbonyl derivatives from a selection of chemotherapeutics, antibiotics, and other pharmaceuticals.

## 4. Enantioselective Decarboxylative Protonation

Enantioselective protonation is a very common process in biosynthetic transformations. Recently, several chemical methods for achieving enantioselective protonation have been developed, employing a variety of precursors and protocols [31]. Clearly, the generation of tertiary carbon stereocenters by using the very small proton atom is a very interesting and attractive process; however, it is also very challenging, because it normally involves undesired racemization reactions. The enantioselective protonation of enolates, using the combination of palladium and organocatalysis, has mainly focused on the generation of the enolate *via* decarboxylation of β-ketoesters [32,33]. An enantioselective decarboxylative protonation can be achieved either generating a chiral Brønsted base (*i.e.*, a chiral metal enolate), or by protonation of an achiral enolate by a chiral proton source. The formation of the enolate in the case of Pd-catalyzed reactions is typically achieved by decarboxylation of benzyl or allyl β-ketoesters.

**Scheme 8:**
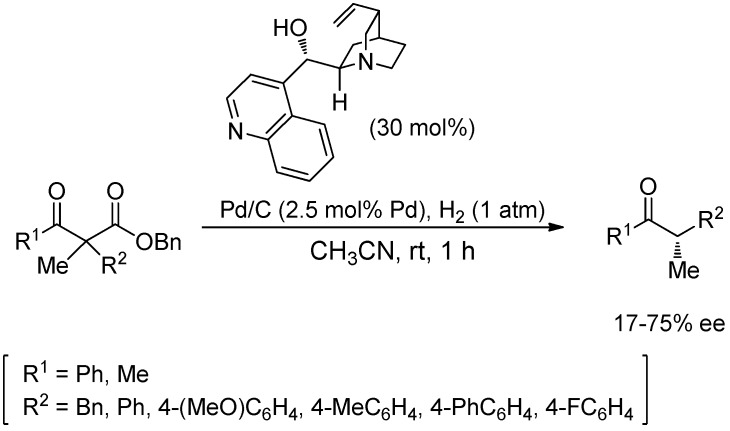
Enantioselective protonation of a linear achiral enolate by a chiral amino alcohol.

Hénin, Muzart, and co-workers have studied the enantioselective decarboxylative protonation of benzyl β-ketoesters to generate an achiral enol that is then protonated by a chiral amino alcohol [34,35,36,37,38]. Usually, moderate enantioselectivities (17%–75%) are obtained for the protonation of both cyclic (which generate stereodefined proton acceptors) and acyclic benzyl β-ketoesters under the optimized conditions (Scheme 8). With respect to the reaction mechanism, Baiker *et al*. have demonstrated that only the debenzylation reaction to afford the corresponding β-ketoacid takes place on the Pd surface, whilst the decarboxylation is a homogeneous process, organocatalyzed by the chiral amino alcohol [39].

**Scheme 9:**
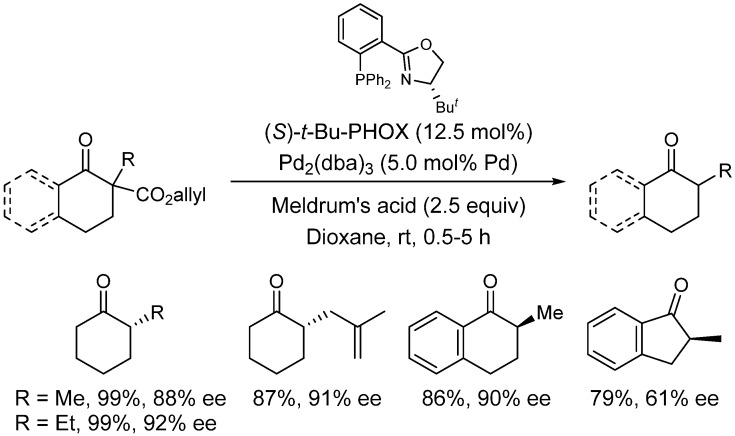
Enantioselective protonation of a chiral enolate by Meldrum’s acid.

The enantioselective protonation by chiral Brønsted acids is strongly dependent on the reaction conditions, thus limiting the scope and applicability of the process. Recently, Stoltz *et al*. have studied the decarboxylative enantioselective protonation of cyclic allyl β-ketoesters [40,41]. In this case, the corresponding chiral enolates are protonated with an achiral proton source, such as Meldrum’s acid [41]. As depicted in Scheme 9, a wide range of tertiary-substituted cyclic ketones can be prepared in very good yields and enantioselectivities using the chiral phosphinooxazoline (PHOX) as ligand for the palladium catalyst [Pd(OAc)_2_].

## 5. Cascade Iminium/Enamine-Palladium Cooperative Catalysis

**Scheme 10:**
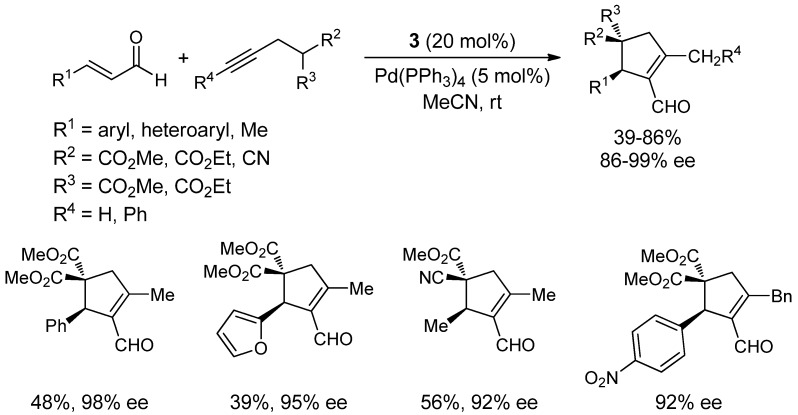
Amine- and palladium-catalyzed enantioselective cycloisomerization.

Lately, remarkable efforts have been made into developing stereocontrolled sequential, cascade or domino reactions, since they allow the syntheses of complex chiral molecules from relatively simple starting materials through multiple consecutive catalytic cycles in an atom-economy fashion [42,43,44]. In this regard, the combination of iminium/enamine and palladium catalysis has been used by different authors to prepare optically active highly functionalized carbo- and heterocycles [45,46,47,48]. After Dixon’s decisive work using copper catalysis [45], Córdova *et al*. reported a simple and highly enantioselective dynamic kinetic asymmetric transformation (DYKAT) involving catalytic iminium activation, enamine activation, and palladium-catalyzed enyne cycloisomerization [46]. Córdova’s methodology allows the preparation of functionalized optically active cyclopentenes with all-carbon quaternary stereocenters in good diastereo- and high enantioselectivities from simple starting materials such as α,β-unsaturated aldehydes (aromatic and aliphatic) and activated propargylated methylenes (Scheme 10). Both terminal and internal alkynes are tolerated under the optimized reaction conditions.

**Scheme 11:**
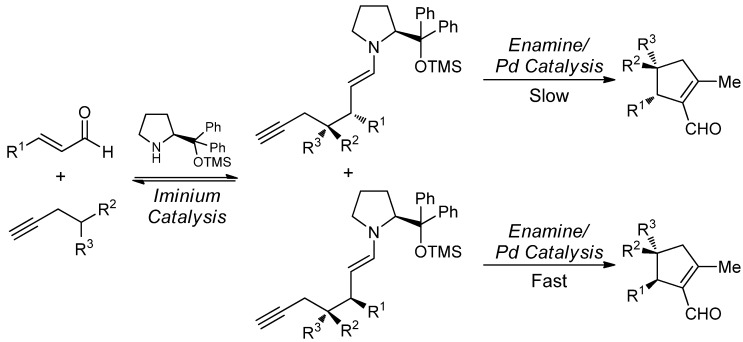
Proposed mechanism for the amine/palladium-catalyzed enantioselective cycloisomerization.

The reaction mechanism involves an initial Michael addition of the activated propargylated methylene derivative to the chiral iminium salt formed between the Jørgensen-Hayashi catalyst and the enal. This reversible addition generates a diastereomeric mixture of Michael adducts which undergoes, at different rates, a Pd(0)/enamine catalyzed cycloisomerization to afford the corresponding products (Scheme 11).

**Figure 6:**
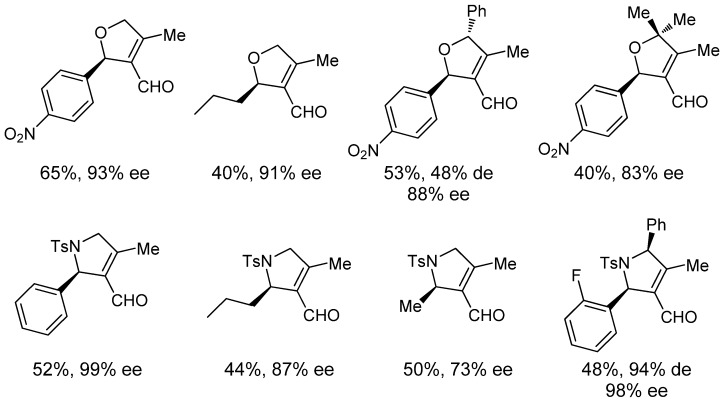
Representative examples of chiral dihydrofurans and pyrrolines prepared by amine- and palladium-catalyzed enantioselective cycloisomerization.

In parallel, chiral dihydrofurans and dihydropyrroles have been prepared using a similar iminium/enamine-palladium cooperative catalytic strategy [47,48]. Thus, Zhao and Córdova have reported a highly enantioselective domino oxa-Michael/carbocyclization between propargyl alcohols and enals to afford the corresponding chiral dihydrofurans in good to high yields and very high enantioselectivities (Figure 6) [47]. Similarly, the reaction between *N*-tosyl propargylamines and enals provides highly functionalized optically active pyrrolines, as depicted in Figure 6, for selected examples [48]. In these two studies, aromatic enals lead to higher enantioselectivities than aliphatic ones. Also, it is worthy to note the ability of the catalytic system towards the synthesis of 2,3,4,5-tetrasubstituted dihydrofurans and pyrrolines, which can be prepared in moderate yield and diastereoselectivity but with high enantioselectivity, by using the corresponding 2-secondary and tertiary propargylic nucleophile (Figure 6). On the contrary, very low yields have been reported when using internal alkynes as nucleophiles.

**Scheme 12:**
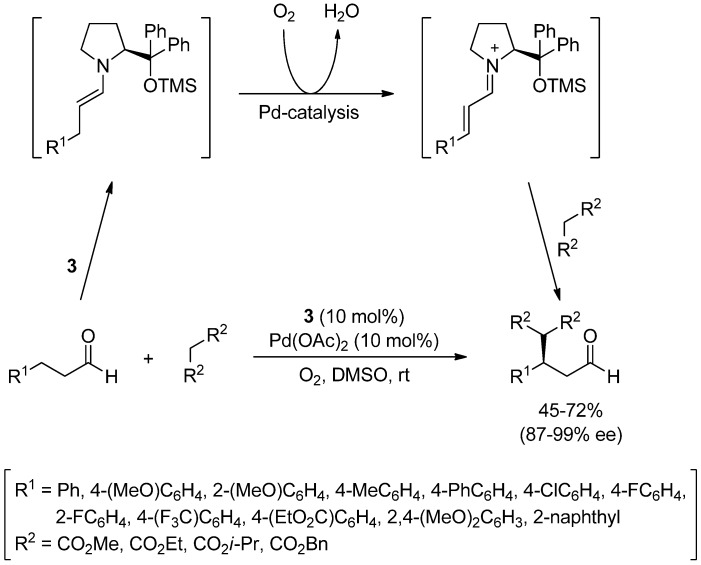
Asymmetric β-functionalization of aldehydes by combination of organocatalysis and palladium catalytic oxidation.

Finally, the enantioselective β-functionalization of aldehydes has been recently achieved by co-catalysis, employing pyrrolidine **3** and palladium acetate. The process involves the formation of the corresponding enamine by reaction of the aldehyde with **3**, followed by the palladium-catalyzed oxidation to the α,β-unsaturated iminium intermediate, which undergoes a Michael addition with diethyl malonate (Scheme 12) [49]. The final products are obtained in moderate to good yields (45%–72%) and with high enantioselectivities (87%–99%).

## 6. Conclusions

The dual activation of simple substrates using the combination of organocatalysis and palladium catalysis has been recently applied to the synthesis of chiral molecules. This distinctive combination brings new opportunities in terms of both selectivity and reactivity, by allowing novel transformations that cannot be performed by using metal catalysis or organocatalysis independently. Pioneering work has been recently reported on the asymmetric α-allylation of carbonyl compounds [17,18,19,20,21,22,23,24,25,26,27], α-fluorination of acyl derivatives [28,29,30], decarboxylative protonation of β-dicarbonyls [32,33,34,35,36,37,38,39,40,41], cyclization reactions of alkynyl carbonyl compounds [46,47,48] and β-functionalization of aldehydes [49], employing this double-catalytic methodology. These reports evidence the versatility of this emerging strategy; however, there is still room for improvement and expansion on the areas of application, as well as on the better understanding of the processes.
